# Complementary and alternative medicine use by diabetes patients in Kerala, India

**DOI:** 10.1017/gheg.2017.6

**Published:** 2017-05-15

**Authors:** N. Vishnu, G. K. Mini, K. R. Thankappan

**Affiliations:** 1 Achutha Menon Centre for Health Science Studies (AMCHSS), Sree Chitra Tirunal Institute for Medical Sciences and Technology (SCTIMST), Trivandrum, India; 2 Centre for Public Health, Amrita Institute of Medical Sciences (AIMS), Amrita Vishwa Vidyapeetham University, School of Medicine, Health Science Campus, Kochi, Kerala, India

**Keywords:** Alternative medicine, diabetes patients, India, Kerala

## Abstract

The study assessed: (1) the prevalence of exclusive use of complementary and alternative medicine (CAM), exclusive use of modern medicine and combined use; (2) the factors associated with exclusive CAM use; and (3) the expenditure for CAM use among type-2 diabetes patients in rural Kerala. We surveyed 400 diabetes patients selected by multi-stage cluster sampling. Exclusive CAM use was reported by 9%, exclusive modern medicine by 61% and combined use by 30%. Patients without any co-morbidity were four times, those having regular income were three times and those who reported regular exercise were three times more likely to use exclusive CAM compared with their counterparts. Expense for medicines was not significantly different for CAM compared with modern medicine both in government and private sector. Patients with any co-morbidity were less likely to use CAM indicating that CAM use was limited to milder cases of diabetes.

## Introduction

India is the second largest country in the world after China with an estimated 69.2 million adults with type-2 diabetes [1]. It is a major public health problem that requires regular medication along with lifestyle modification in order to achieve adequate control. In developing countries such as India, access to modern medicine is limited in the public sector and patients usually approach private sector including all systems of medicine. Previous studies have reported that patients with diabetes were more likely to use complementary and alternative medicine (CAM) compared with other patient groups [2]. The major reasons for using CAM for the treatment of diabetes were fear about side effects, dissatisfaction with healthcare providers and higher costs of modern medicine [3–5]. Other reasons were higher level of medication adherence along with better understanding of the need for lifestyle changes for diabetes management during CAM treatment [6] and easy availability of CAM without the prescription of a doctor [7].

The use of CAM for patients with diabetes was reported to be common in almost all parts of the world [8–10]. However, different definitions were used for CAM, which was one of the reasons for a wide range of prevalence of CAM use ranging from 17% to 73% [11]. CAM use prevalence in the USA ranged from 31% to 57% among diabetes patients [12], 63% in Bahrain [13], 62% in Mexico [14], 17% in UK [15] and 25% in Canada [16]. China had a long tradition of use of herbal medicine for diabetes. The findings of a systematic review reported that Chinese herbal medicines were reported to be more effective for diabetes compared with lifestyle modification alone [17]. In China, traditional medicines accounts for 40% of all healthcare delivered [18].

A few studies reported CAM use from different parts of India. One such study from the state of Uttar Pradesh reported a prevalence of 68% CAM use among diabetes patients [19]. CAM use for selected chronic diseases (HIV, epilepsy, rheumatoid arthritis and diabetes) in India was reported to be 35% with the highest use of CAM among diabetes patients (63.2%) in Maharashtra [20]. India has a rich tradition of use of ayurvedic medicines and has a government department for CAM which is named as ‘AYUSH’ (Ayurveda, Yoga, Naturopathy, Unani, Sidha and Homeopathy). Within India, Kerala state reported the highest prevalence of type-2 diabetes (20.6%) in rural areas [21]. Adherence to modern medicines among diabetes patients using Morisky's scale in rural Kerala was reported to be 26% [22] indicating a probability of higher use of CAM in the state. However, only one study was reported form Kerala on CAM use [23] based on a convenient sample of 50 diabetes patients from urban areas. The objectives of the present study were to find out: (1) the prevalence of exclusive use of CAM, exclusive use of modern medicine and combined use; (2) the factors associated with exclusive CAM use; and (3) the expenditure for CAM use among type-2 diabetic patients in rural Kerala.

## Methods

We conducted a community based cross-sectional study among self-reported diabetes patients in rural Kollam district of the Indian state of Kerala during June to September, 2015. All the 14 districts in the state were assigned numbers from 1 to 14 from north to south and one computer-generated random number was selected. That number corresponded to the district of Kollam. With an anticipated prevalence of 20% CAM use among diabetes patients [23], with 95% confidence level and a precision of 5% the sample size was calculated as 246. As it was a cluster sampling, a design effect of 1.5 was used and the sample size of 369 thus obtained was rounded off to 400.

**Fig. 1.**
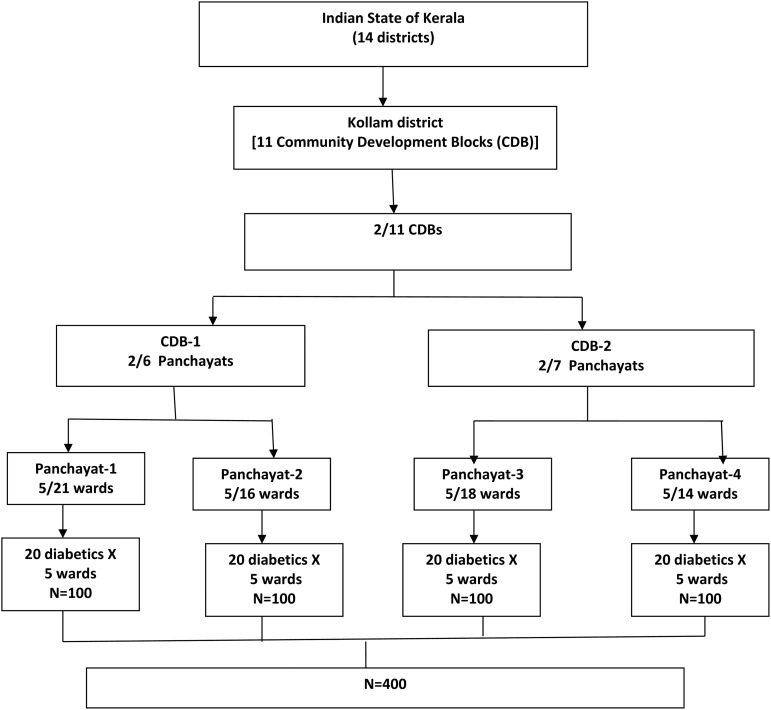
Sample selection process.

There were 11 community development blocks (CDBs) in Kollam district, out of which two CDBs were randomly selected. From each of these CDB, two *Panchayats* each were selected randomly. Five wards (ward is the smallest geographical unit of the decentralized government in Kerala) were randomly selected from each Panchayat. From each ward, 20 self-reported diabetes patients were identified for the study making a total sample of 400 (see Fig. 1).

We started the survey in each selected ward by locating the center of the ward, after which the first house was identified as per the guidelines of the World Health Organization (WHO) expanded program on immunization cluster sampling technique [24]. Subsequent households were identified in a clockwise direction till we got 20 patients from each ward. Self-reported diabetes patients aged 18 years and above were selected from each household based on the diagnosis of diabetes by a modern medical practitioner. Patients who did not understand or speak *Malayalam* (local language) and those who did not consent (only one patient) were excluded from the study.

Data were collected using a pre-tested structured interview schedule. Information on age, sex, education, employment, household average monthly expenditure, presence of co-morbidity, use of any specific diet for control of diabetes, practice of regular exercise for diabetes control, system of medicine used and monthly expenditure for medicine was collected. We decided to include retired patients and pensioners in the ‘employed’ group since they were getting income on a regular basis similar to those who were employed. Therefore, we grouped them into a single category of having regular income for analysis.

We asked a question on the presence of any co-morbidity such as cardiovascular disease, elevated cholesterol, kidney disorders, neurological problem, high blood pressure, vision impairment, sexual dysfunction, liver disease and other non-communicable diseases. The presence of any of these diseases was considered as ‘presence of co-morbidity’.

CAM use for our study was defined as use of alternative medicines or treatment practices, including ayurveda, homeopathy, unani, siddha, yoga and naturopathy, acupuncture, reiki (a healing technique by means of touch) and herbal medicines. Herbal medicines are those that are believed to be beneficial for the management of diabetes by the people. Herbal medicines include herbs, herbal materials, herbal preparations and finished herbal products that contain parts of plants, or other plant materials, or combinations.

Ethical clearance for the study was obtained from the institute ethics committee of Sree Chitra Tirunal Institute for Medical Sciences and Technology, Trivandrum. Informed written consent was obtained from all participants before the study.

All statistical analysis were done using SPSS version 17 (SPSS Inc., Chicago, Illinois). Bivariate analysis was done using χ^2^ test for categorical variables. Multiple logistic regression analysis was done to find out the correlates of exclusive CAM use. In multivariate analysis, the sample was divided into two: those with any co- morbidity and those without. Median expense for medicine was compared between CAM and modern medicine both in public and private sector using Mann–Whitney test. A ‘*p*’ value of <0.05 was used as cut-off for statistical significance.

## Results

Mean age of the sample was 59 years (s.d.: 10, range: 29–87) and 54.5% were men. Eighty-four percent were currently married and 69% had less than higher secondary education. Thirty-six percent were having regular income of which, 10.6% were government employees, 47.9% were private employees and the remaining 41.5% were self-employed, retired or pensioners. On an average there were four members in the household. The monthly median expenditure of the household was INR 10 000 (~USD 149).

Average duration of diabetes was 8 years. Forty-eight percent of patients visited their doctor in the last month, 33% between 1 and 6 months, 9% between 6 months to 1 year and 10% visited more than a year ago. More than half of the sample (59%) sought treatment for diabetes from private healthcare institutions and the remaining from government healthcare institutions.

Majority of them (87%) were diagnosed with diabetes during medical consultation for other disease, 11% during regular checkup and 2% in other occasions such as medical camps and medical checkup for visa application. About 85% were following a specific diet pattern and 33% exercised regularly for the control of diabetes.

A substantial number of our diabetes patients reported the presence of co-morbidities such as vision impairment (*n* = 260, 65%), elevated cholesterol (*n* = 222, 56%), sexual dysfunction (*n* = 188, 47%), high blood pressure (*n* = 143, 36%), cardio vascular disease (*n* = 128, 32%), neurological problems (*n* = 42, 11%), kidney disorders (*n* = 27, 7%), liver disease (*n* = 6, 2%) and other diseases such as asthma, hernia, hyperthyroidism and prostrate problem (*n* = 16, 4%).

For the treatment of diabetes 61% used modern medicine only (men 62%, women 60%), 9% CAM use only (men 11%, women 7%) and 30% combination of both (men 27%, women 33%). Average distance to nearest health facility was reported as 2.4 km for modern medicine, 1.9 km for ayurveda and 2.1 km for homeopathy. Background characteristics by CAM use are presented in Table 1. Different types of CAM use by sample participants are presented in Table 2. None of the patients reported the use of unani, siddha, naturopathy, acupuncture or reiki.

**Table 1.** Background characteristics by CAM use

Variables	CAM users	CAM non-users	Overall
Age			
<55	61(42.1)	84(57.9)	145(100.0)
55–65	62(40.5)	91(59.5)	153(100.0)
>65	32(31.4)	70(68.6)	102(100.0)
Sex			
Men	82(37.6)	136(62.4)	218(100.0)
Women	73(40.1)	109(59.9)	182(100.0)
Education			
<higher secondary	106(38.5)	169(61.5)	275(100.0)
≧higher secondary	49(39.2)	76(60.8)	125(100.0)
Monthly average expenditure			
<10 000	50(35.7)	90(64.3)	140(100.0)
≧10 000	105(40.4)	155(59.6)	260(100.0)
Having regular income			
Yes	66(34.5)	76(53.5)	142(100.0)
No	89(34.5)	169(65.5)	258(100.0)
Type of hospital			
Government	67(41.1)	96(58.9)	163(100.0)
Private	88(37.1)	149(62.9)	237(100.0)
Following specific diet for the control of diabetes			
No	22(37.3)	37(62.7)	59(100.0)
Yes	133(39.0)	208(61.0)	341(100.0)
Doing regular exercise for control of diabetes			
No	90(33.5)	179(66.5)	269(100.0)
Yes	65(49.6)	66(50.4)	131(100.0)
Presence of co-morbidity			
No	19(57.6)	14(42.4)	33(100.0)
Yes	136(37.1)	231(62.9)	367(100.0)
Duration of diabetes			
<8 years	95(40.1)	142(59.9)	237(100.0)
≧8 years	60(36.8)	103(63.2)	163(100.0)

**Table 2.** Proportion (%) of different types of CAM use

Type of CAM used for diabetes	Proportion (%)^a^ *N* = 155
Ayurveda	26.7
Homeopathy	3.2
Yoga	7.1
Herbal medicine	63.2
Sugar therapy^b^	2.6

a Total do not add to 100% due to multiple use.

b A type of treatment offered by certain medical practitioners combining sugary food along with advice for regular exercise for management of diabetes patients in Kerala.

Among CAM users, 18% had their relatives, friends or neighbors working in healthcare institutions providing CAM. Multiple reasons were reported for using CAM for the treatment of diabetes: modern medicine treatment was not effective (37%) and too toxic (13%), CAM use was user friendly (24%), free from adverse effects (21%), available easily (9%) and low cost (1%). Thirty-nine percent consulted a doctor for getting CAM treatment. Among those who consulted, 39% consulted only once.

More than half of the CAM users (57%) used it daily, 8% weekly, 32% occasionally and 3% only once previously. The main source of getting CAM was CAM practitioners (46%), friends and relatives (7%) and the remaining 47% from other sources such as neighbors and locally available herbal medicines. Thirteen of the CAM users (8%) reported that they experienced some side effects. More than half (52%) recommended CAM for someone with diabetes. Eighty-seven percent reported some relief in diabetes symptoms after the use of CAM and 71% were satisfied with the use of CAM.

The median expense for modern medicine was almost similar in government (INR 233) and private sector (INR 252). Among the CAM users, 63.2% used herbal medicine and 7.1% used yoga, which did not cost any money. Hundred and one out of the 155 CAM users (65.2%) did not report any expenses. For CAM also the median expense was similar in government (INR150) and private sector (INR 187). The median expense for CAM was similar to the median expense for modern medicine in the government sector (*p* > 0.05). Similarly the median expense for CAM was similar to the median expense for modern medicine in the private sector (*p* > 0.05).

The variables found significantly associated with exclusive CAM use in the bivariate analysis were put as independent variables in multivariate analysis. The age adjusted logistic regression analysis results are presented in Table 3. CAM use was higher among those having no co-morbidity (OR 4.19, 95% CI 1.14–12.42), those having regular income (OR 2.84, CI 1.28–6.27) and those reporting regular exercise for control of diabetes (OR 2.60, CI 1.24–5.45).

**Table 3.** Results of age adjusted multiple logistic regression analysis of correlates of exclusive CAM use

Variable	Exclusive CAM use (%)	OR (95% CI)
Any co-morbidity		
Yes	11.5	Reference
No	33.3	4.19 (1.14–12.42)
Doing regular exercise for control of DM		
No	8.7	Reference
Yes	23.3	2.60 (1.24–5.45)
Having regular income		
No	8.6	Reference
Yes	21.6	2.84 (1.28–6.27)

## Discussion

Proportion of patients who reported using exclusive CAM for diabetes was 9%, whereas patients who used CAM along with modern medicine were 30% and those who used modern system of medicine exclusively were 61%. Studies on exclusive CAM use are limited since combined use of CAM with modern medicine is what is practiced in many developed countries. The previous study from urban Kerala using a small sample of 50 diabetes patients [23] reported a prevalence of 20%, which was lower than the present study result of 39%. The higher use of CAM in our study might be due to limited availability of free and regular supply of modern medicine for diabetes from the public health system in Kerala [25]. Patients were complementing CAM with modern medicines probably due to the cultural practices, lack of perceived side effects, easy availability and acceptability of CAM in the state.

The use of CAM in our study (39%) was lower than that reported among diabetes patients in Mumbai (63%) [20]. Use of CAM was reported to range from 25% to 85% among Turkey diabetes patients [26–28]. Higher CAM use was reported among diabetes patients in Bahrain [13], in Mexico [14], Taiwan [29] and Palestinian [30] and lower prevalence of CAM use in Jordan [31], UK [15] and in Canada [16].

The present study participants reported that 39% consulted CAM practitioners which was higher the than 7% reported from Lebanon [6] and 29% reported from Maharashtra [20]. The low level of physician consultation on CAM use is likely to result in drug interactions. Considering the frequent use of CAM by diabetes patients, doctors treating them should be aware of CAM use and they should ask their patients about the CAM use. This is important in the context of high level of refusing consent by CAM users (59.9) in participating in a previous study mainly because of the reason that they did not want to reveal CAM use to their physicians [20].

Earlier studies reported high use of CAM among women compared with men [32, 33]. This difference was not seen in our study where CAM use was similar among men and women.

Patients without any co-morbidity were four times more likely to use exclusive CAM compared with patients with co-morbidity indicating that exclusive CAM use was preferred by milder or patients without co-morbidities. There is a possibility that these patients may be afraid of the side effects of modern medicine and a perception of comparatively safer feeling toward CAM. The likely users of CAM were those without any co-morbidity and in early stage of disease, although the latter finding was not statistically significant.

Another major factor associated with exclusive CAM use was the practice of regular exercise for the control of diabetes. A similar finding was reported among Australian women [34]. CAM use was a significant predictor of physical activity among breast cancer survivors [35]. Those who are likely to involve in lifestyle modification would like to control disease without ‘strong’ medications such as modern medicine. Many people in Kerala are afraid of the side effects of modern medicine resulting in either shifting to alternative medicine or combining modern medicine with alternative medicine.

The third major factor associated with exclusive CAM use was employment status of patients. Unlike what was reported earlier [6], our finding showed that patients who were having regular income were three times more likely to use exclusive CAM compared with those who did not have regular income. This could be due to the peer influence at work places.

Our finding on the expenses for medicine was contrary to our expectation that the median expense for CAM in both government and private sector was similar to that of modern medicine. One of the reasons for CAM use was reported to be the low cost of CAM. However, our data showed that the cost was similar. More than 50% of our patients used herbal medicines, which did not cost any money. Therefore the perception of low cost applies only to those who are using CAM other than ayurveda and homeo.

As per the reported monthly expenditure for modern medicine, the annual expenses for medicine for one patient was estimated (expenditure in the last month multiplied by 12) to be INR 2796 (~USD 42) in the public sector and in the private sector INR 3024 (~USD 45). The annual expenditure for a diabetic patient, irrespective of the sector from which they were accessing the services, was around rupees 3000 (~USD 45) per year. As per the Census 2011 data, Kerala had 23.38 million individuals in the age group of 20 years and above. Among them, at least 20% are likely to have diabetes (4.7 million) [21]. The total drug expenses for these diabetes patients will be INR 14 100 million (~USD 210 million). The total government health expenditure including medical education, health services and AYUSH for Kerala in the financial year 2014–2015 was INR 49 774 million (~USD 741 million) [36]. If all the diabetes patients in Kerala are given free medicines, government will have to spend 28.7% of state's total health expenditure. It is therefore important to prevent or delay the onset of diabetes in Kerala by implementing effective community based intervention programs for enhancing physical activity and promoting healthy eating behavior among the entire population. The Kerala Diabetes Prevention Program (KDPP), a cluster randomized controlled trial, has shown that such interventions are feasible and effective [37]. Since these programs are addressing major risk factors of diabetes such as physical inactivity, unhealthy diet, tobacco use, alcohol use, etc. such programs are likely to reduce other non-communicable diseases also since the risk factors are common for major NCDs.

One of the limitations of our study was self-reported information. The findings of our study may not be generalizable to the entire state since we studied only one district and did not use sampling weight for analysis. Kerala is generally known for its Ayurveda traditions and the results are likely to be not representative of India.

## Conclusion

Patients without any co-morbidity, patients who were having regular income and patients who reported regular exercise were significantly more likely to use exclusive CAM compared with their counterparts. Expense for medicines was not significantly different for CAM compared with modern medicine both in government and private sector. If all the diabetes patients in Kerala are given free medicines, government will have to spend 28.7% of state's total health expenditure on diabetes medicines alone. It is therefore important to prevent or delay the onset of diabetes in Kerala by implementing effective community-based intervention programs for enhancing physical activity and promoting healthy eating behavior among the entire population.
